# Inhibitory Effects of *Colocasia esculenta* (L.) Schott Constituents on Aldose Reductase

**DOI:** 10.3390/molecules190913212

**Published:** 2014-08-27

**Authors:** Hong Mei Li, Seung Hwan Hwang, Beom Goo Kang, Jae Seung Hong, Soon Sung Lim

**Affiliations:** 1 Department of Pharmacology, College of Medicine, Hallym University, 200-702 Chuncheon, Korea; E-Mail: lihm@hallym.ac.kr; 2 Department of Food and Nutrition, Hallym University, 200-702 Chuncheon, Korea; E-Mails: isohsh@gmail.com (S.H.H.); kbgda87@naver.com (B.G.K.); 3 Department of Physical Education, Hallym University, 200-702 Chuncheon, Korea; E-Mail: jayshong@hallym.ac.kr; 4 Department of Center for Aging and Health Care, Hallym University, 200-702 Chuncheon, Korea

**Keywords:** *Colocasia esculenta* (L.) Schott, aldose reductase, antioxidant, diabetic complication

## Abstract

The goal of this study was to determine the rat lens aldose reductase-inhibitory effects of 95% ethanol extracts from the leaves of *C. esculenta* and, its organic solvent soluble fractions, including the dichloromethane (CH_2_Cl_2_), ethyl acetate (EtOAc), *n*-butanol (BuOH) and water (H_2_O) layers, using dl-glyceraldehyde as a substrate. Ten compounds, namely tryptophan (**1**), orientin (**2**), isoorientin (**3**), vitexin (**4**), isovitexin (**5**), luteolin-7-*O*-glucoside (**6**), luteolin-7-*O*-rutinoside (**7**), rosmarinic acid (**8**), 1-*O*-feruloyl-d-glucoside (**9**) and 1-*O*-caffeoyl-d-glucoside (**10**) were isolated from the EtOAc and BuOH fractions of *C. esculenta*. The structures of compounds **1**–**10** were elucidated by spectroscopic methods and comparison with previous reports. All the isolates were subjected to an *in vitro* bioassay to evaluate their inhibitory activity against rat lens aldose reductase. Among tested compounds, compounds **2** and **3** significantly inhibited rat lens aldose reductase, with IC_50_ values of 1.65 and 1.92 μM, respectively. Notably, the inhibitory activity of orientin was 3.9 times greater than that of the positive control, quercetin (4.12 μM). However, the isolated compounds showed only moderate ABTS^+^ [2,29-azinobis-(3-ethylbenzothiazoline-6-sulfonic acid)] activity. These results suggest that flavonoid derivatives from *Colocasia esculenta* (L.) Schott represent potential compounds for the prevention and/or treatment of diabetic complications.

## 1. Introduction

According to the World Health Organization (WHO), approximately 200 million people worldwide suffer from diabetes, and it is estimated that this disease will have a severe impact on human health by 2025 [1]. Aldose reductase (AR) is a key enzyme in the polyol pathway that plays important roles in cataract formation and the pathogenesis of diabetic complications such as neuropathy, nephropathy, and retinopathy [2]. Therefore, there is a growing interest in searching for drugs to alleviate the symptoms of diabetic complications. AR is an NADPH-dependent oxidoreductase and it is an important enzyme in the polyol pathway, which catalyses the reduction of glucose to sorbitol, which is further metabolized to fructose by sorbitol dehydrogenase [3]. Thus, AR inhibition represents a key point for the prevention and attention of long-term diabetic complications [4].

As an abundant source of bioactive chemicals plants are an important resource for the development of new drugs [5]. Many flavonoids and polyphenols effectively inhibit AR [6,7,8], and in most cases, herbal medicines lack toxicity and side effects [9,10,11]. Oxidative stress contributes to the progression of diabetes and its complications. Diabetes is usually accompanied by free radical production and impaired antioxidant defenses [12].

*Colocasia esculenta* (L.) Schott, commonly known as taro, is a tropical perennial plant that is native to Asia and the Pacific, and widely distributed in tropical latitudes [13]. It is a starchy root crop with wide leaves, that are edible. Taro is the main food source for approximately 500 million people living in Asia, Africa, Middle America, and the Pacific Islands [14]. *C. esculenta* is reported to display anti-diabetic, anti-inflammatory, anti-oxidant and anti-cancer activities [15]. Studies on the chemical constituents of *C. esculenta* have reported the presence of pelargonidin-3-glucoside, cyanindin-3-rhamnoside, cyanidin-3-glucoside, orientin, isoorientin, vitexin, isovitexin and luteoin-7-*O*-sophoroside [16]. However, no studies have investigated the AR inhibitory activity constituents from *C. esculenta* leaves.

The aim of this study was to investigate the *in vitro* inhibitory effects of *Colocasia esculenta* (L.) Schott extract and its isolated constituent on AR enzyme activity. We assessed the ability of the major compounds to decrease galactitol accumulation in the lens of a galactosemic rat model *ex vivo* and their antioxidant effects. 

## 2. Results and Discussion

In most cases, natural herbal medicines lack toxic and side effects, so there is growing interest in natural products as sources of new drugs [17]. For years, many medicinal plants and their extracts have been demonstrated to effectively treat diabetes [18]. The purpose of this study was to identify new AR inhibitors (ARI) from *Colocasia esculenta* (L.) Schott for the treatment of diabetic complications.

A 95% ethanol extract of *C. esculenta* leaves was found to exhibited inhibitory activity against crude rat lense aldose reductase (rAR). Consequently, the 95% ethanol extract of *C. esculenta* leaves was further partitioned by systematic fractionation. Among the resulting fractions, the ethyl acetate (EtOAc) and *n*-butanol (BuOH) soluble fractions exhibited potent inhibitory activity against rAR with IC_50_ values of 2.07 and 5.05 μg/mL, respectively, compared with the positive control quercetin IC_50_ = 1.09 μg/mL (Table 1). Therefore, this study focused on the isolation of ARI compounds from these fractions.

**Table 1:** Inhibitory effects of the *Colocasia esculenta* (L.) Schott on rat lens aldose reductase (rAR).

Extract and Fraction	Concentration (µg/mL)	Aldose Reductase Inhibition (%)	IC_50_ (μg/mL)
95% EtOH extract	10	43.66 ± 0.41	-
Hexane fr.	10	29.11 ± 0.31	-
CHCl_3_ fr.	10	13.05 ± 0.12	-
EtOAc fr.	5	74.89 ± 1.17	2.07 ± 0.02
	2.5	54.83 ± 1.27	
	1	30.78 ± 0.64	
*n*-BuOH fr.	10	84.02 ± 0.97	5.05 ± 0.55
	5	51.57 ± 0.53	
	1	20.33 ± 1.15	
Water fr.	10	24.3 ± 0.64	-
Quercetin ^a^	2.5	69.64 ± 0.72	1.09 ± 0.01
	1	48.9 ± 0.51	
	0.5	31.62 ± 0.09	

Inhibition rate were calculated as percentages with respect to the control value. ^a^ Quercetin was used as positive control. Inhibitory effect was expressed as mean ± SD (standard deviation) of triplicate experiments.

**Figure 1:**
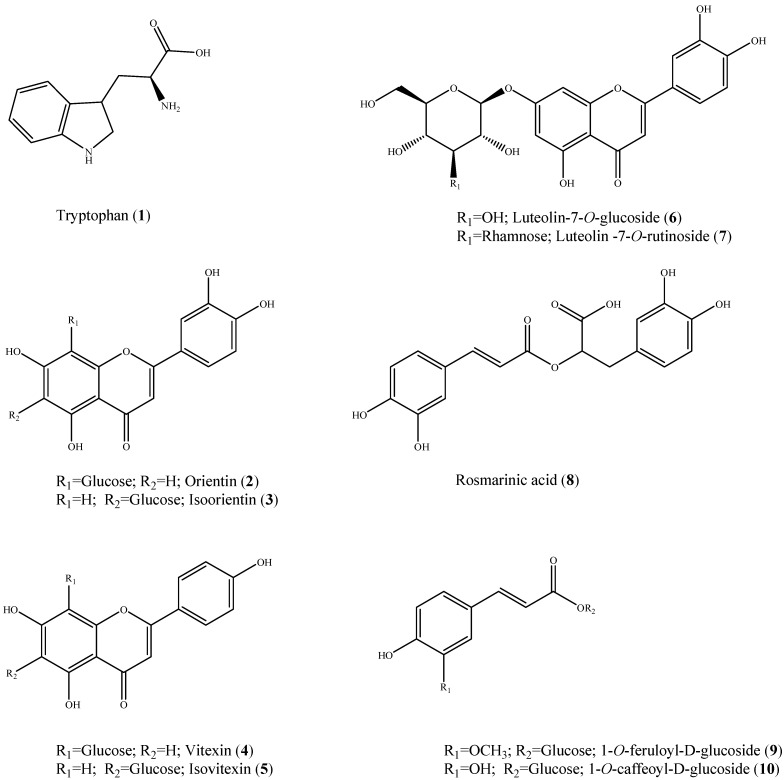
Structures of the constituents isolated from *Colocasia esculenta* (L.) Schott.

The EtOAc and BuOH soluble fractions were isolated using Sephadex LH-20 column chromatography, and the resulting components were identified as tryptophan (**1**) [19], orientin (**2**) [20], isoorientin (**3**) [20], vitexin (**4**) [20], isovitexin (**5**) [20], cynaroside (**6**) [21], lonicerin (**7**) [22], rosmarinic acid (**8**) [23], 1-*O*-feruloyl-d-glucoside (**9**) [24] and 1-*O*-caffeoyl-d-glucoside (**10**) [24]. The structures of these compounds (Figure 1) were elucidated based on 1D and 2D (HMQC and HMBC) NMR spectral data and by comparison with published spectral data. We compared the ability of the isolated compounds and quercetin, a positive control, to inhibit rAR activity (Table 2). Among the isolated constituents, compounds **2** and **3** displayed potent rAR inhibitory activity, with IC_50_ values of 1.65 and 1.92 µM, respectively. Compound **7** has an IC_50_ value of 2.59 µM, which was also higher than that of quercetin (IC_50_ = 4.12 µM). Compounds **5**, **6** and **10** have IC_50_ values of 7.36, 7.47 and 14.14 µM, respectively. Jung *et al.* reported that orientin and isoorientin isolated from *Phyllostachys nigra* showed were potent AR inhibitors and advanced glycation end products [25]. Several possible relationships may exist between the structure and inhibitory activity of flavones, since hydroxylation in the 4'-position has beneficial effects and the double bond between C-2 and C-3 increases the inhibitory activity [26]. The aldose reductase inhibitory activity of compounds containing a catechol moiety in the B ring was greater than in those with a 4'-hydroxy group [27].

Here, compounds **2**, **3**, **7** and quercetin are all exhibited greater activity than vitexin and isovitexin (Table 2). The inhibitory activity of 8-C-glucosyl 3',4',5,7-tetrahydroxy flavone was a slightly stronger than that of 6-C-glucosyl [25]. 6-C-glucosyl and 8-C-glucosyl flavone showed more potent activity than the 7-C-glucosyl flavone.

Flavonoids are reported to dramatically inhibit aldose reductase. In particular, quercetin, quercitrin, and myricitrin are lead compounds that preceded the discovery of tolrestat. In order to confirm the type of rat aldose reductase inhibitory activity caused by compounds **2** and **3**, we performed a kinetic study using different concentrations of dl-glyceraldehyde as a substrate (concentration: 0.1–1 mM).

**Table 2:** Inhibitory effects of the constituents isolated from the *Colocasia esculenta* (L.) Schott on rat lens aldose reductase (rAR).

Compounds		Concentration (µg/mL)	Inhibition (%)	IC_50_ (µg/mL)	IC_50_ (µM)
	Quercetin ^a^	5	81.14 ± 0.81	1.25 ± 0.05	4.12 ± 0.16
		1	48.60 ± 1.44		
		0.5	26.32 ± 0.59		
**1**	Tryptophan	10	19.99 ± 2.01	-	-
**2**	Orientin	5	93.97 ± 0.98	0.74 ± 0.03	1.65 ± 0.07
		1	64.57 ± 0.52		
		0.5	35.29 ± 0.36		
**3**	Isoorientin	5	77.73 ± 0.77	0.86 ± 0.05	1.92 ± 0.11
		1	57.64 ± 0.58		
		0.5	37.23 ± 0.37		
**4**	Vitexin	10	48.53 ± 0.87	-	-
**5**	Isovitexin	10	76.59 ± 0.74	3.18 ± 0.31	7.36 ± 0.72
		5	60.29 ± 0.61		
		1	23.34 ± 0.25		
**6**	Luteolin-7-*O*-glucoside	10	76.24 ±0.77	3.35 ± 0.32	7.47 ± 0.71
		5	61.88 ± 0.60		
		1	19.06 ± 0.21		
**7**	Luteolin-7-*O*-rutinoside	5	72.69 ± 0.73	1.54 ± 0.14	2.59 ± 0.26
		1	40.31 ± 0.40		
		0.5	29.91 ± 0.30		
**8**	Rosmarinic acid	5	87.33 ± 0.87	2.99 ± 0.27	5.38 ± 0.75
		1	62.79 ± 0.60		
		0.5	18.34 ± 0.19		
**9**	1-*O*-Feruloyl-d-glucoside	10	4.06 ± 0.40	-	-
**10**	1-*O*-Caffeoyl-d-glucoside	10	73.19 ± 0.99	4.84 ± 0.47	14.14 ± 0.14
		5	55.57 ± 0.78		
		2.5	24.72 ± 0.36		

^a^ Quercetin was used as positive control. Inhibition rate was calculated as percentage with respect to the control value. The IC_50_ values of each sample were estimated from the least-squares regression line of the logarithmic concentration plotted against inhibitory activity. Inhibitory effect was expressed as mean ± SD of triplicate experiments.

**Figure 2:**
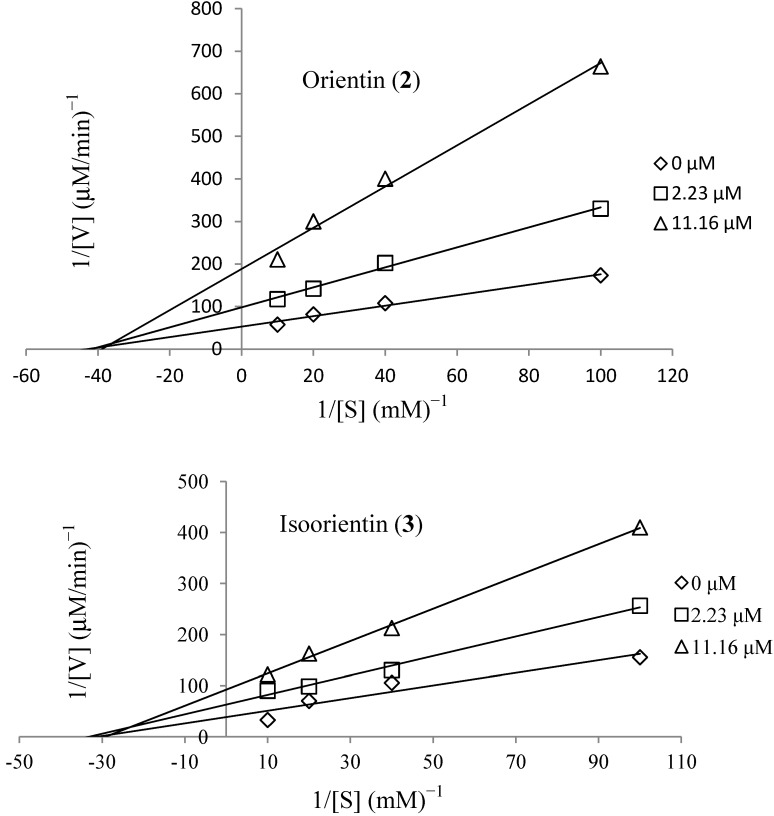
Lineveaver-Burk plots showing the reciprocal of the velocity (1/v) of recombinant rat lens aldose reductase *versus* the reciprocal of the substrate concentration (1/s) with dl-glyceraldehyde as the substrate at concentrations of 0.1 to 1 mM.

Kinetic analysis using Lineweaver-Burk plots of 1/velocity and 1/concentration for compounds **2** and **3** are shown in Figure 2. Changes in substrate concentration resulted in different slopes and *x-axis* intersects for the uninhibited enzyme and different concentrations of the compounds.

The *K_m_* (Michaelis-Menten constant) was unchanged, while the maximum velocity (*V_max_*) decreased. Therefore, compounds **2** and **3** are noncompetitive, indicating that the inhibitor were unable to bind the substrate binding area or the NADPH binding area. Next, we calculated the inhibitory constant (*K_i_*) from secondary Lineweaver-Burk plots, and the compounds display *K_i_* values of 3.23 × 10^−6^ M and 5.88 × 10^−6^ M, respectively. The lower *K_i_* value implies tighter binding with the free enzyme AR or the enzyme substrate complex, so compound **2** is expected to be a more effective inhibitor than compound **3** [28].

In diabetes, activation of the polyol pathway leads to the accumulation of sorbitol in various tissues. Excess glucose causes flux through the polyol pathway, which significantly increase AR activity and the accumulation of sorbitol [29]. Galactose produces a greater accumulation of polyol than glucose, because it has a higher affinity for AR [29,30,31]. Thus, we evaluated the effects of compounds **2** and **3** on galactitol accumulation in rat erythrocytes and lenses. Incubation of rat lenses for 6 days in 30 mM galactose increased the intracellular accumulation of galactitol. As shown in Table 3, compound **2** and compound **3** decreased galactitol accumulation in rat erythrocyte by 16.1% and 35.2%, respectively at 5 µg/mL (quercetin = 47.9%). These compounds also inhibited galactitol accumulation in isolated rat lenses by 9.1% and 2.7% at 5 µg/mL, respectively (quercetin = 3.8%).

**Table 3:** Inhibitory effect of the constituents on the galactitol accumulation in rat lenses and erythrocyte.

Compounds	Rat Erythrocyte Galactitol Content (µM) ^a^	Galactitol Content µg/lens Wet Weight (g) ^b^
Galactitol free	-	3.13 ± 0.06
Control	86.70 ± 1.61	250.47 ± 5.01
Quercetin ^c^	45.17 ± 0.81	241.07 ± 4.57
Isoorientin	56.18 ± 1.00	243.14 ± 4.71
Orientin	72.72 ± 0.98	227.89 ± 4.52

^a^ Erythrocytes were incubated in a Krebs-Ringer bicarbonate buffer containing 30 mM galactose and in the presence or absence of 5 µg/mL compounds. ^b^ Mean of three duplication analyses of rat lens with compounds at a concentration of 5 µg/mL. ^c^ Quercetin was used as positive control. Inhibitory effect was expressed as mean ± SD of triplicate experiments.

Oxidative stress plays a key role in the pathogenesis of vascular complications in diabetes, and it provides an early marker of such damage in the development of endothelial dysfunction [32]. Therefore, we evaluated the antioxidant activity of *C. esculenta* and its isolated constituents. Only the EtOAc fraction showed minor activity in the ABTS^+^ assay (Table 4). The ABTS^+^ inhibitory activities of the isolated constituents were tested, and trolox used as the positive control. Except for compounds **1**, **4** and **5**, all compounds exhibited ABTS^+^ inhibitory activity (Table 5). 

**Table 4:** Antioxidant effects of the *Colocasia esculenta* (L.) Schott on inhibition of the ABTS^+^.

Extract and Fraction		Concentration (µg/mL)	Inhibition (%)	IC_50_ (µg/mL)	IC_50_ (µM)
Trolox ^a^		8.33	92.92 ± 0.06	2.87 ± 0.01	11.49 ± 0.04
		3.33	57.40 ± 2.03		
		1.67	27.07 ± 1.15		
Quercetin ^b^		1.67	81.36 ± 1.75	0.97 ± 0.04	3.20 ± 0.12
		0.83	42.49 ± 3.39		
		0.33	22.98 ± 3.55		
95% EtOH Extract		10	26.07 ± 0.61	-	-
Hexane fr.		33.33	14.77 ± 0.85	-	-
CHCl_3_ fr.		33.33	42.4 ± 1.13	-	-
EtOAc fr.		16.67	71.10 ± 3.62	10.86 ± 0.84	-
		8.33	41.64 ± 3.10		
		3.33	21.78 ± 2.11		
	*n*-BuOH fr.	33.33	48.35 ± 1.07	-	-
	Water fr.	33.33	9.90 ± 0.60	-	-

Inhibition rate were calculated as percentages with respect to the control value. ^a^ Trolox and ^b^ quercetin were used as positive controls. Inhibitory effect was expressed as mean ± SD of triplicate experiments

**Table 5:** Antioxidant effects of the constituents isolated from the *Colocasia esculenta* (L.) Schott on inhibition of the ABTS^+^.

Compounds	Concentration (µg/mL)	Inhibition (%)	IC_50_ (µg/mL)	IC_50_ (µM)
Trolox ^a^	8.33	92.92 ± 0.06	2.87 ± 0.01	11.49 ± 0.04
	3.33	57.40 ± 2.03		
	1.67	27.07 ± 1.15		
Quercetin ^b^	1.67	81.36 ± 1.75	0.97 ± 0.04	3.20 ± 0.12
	0.83	42.49 ± 3.39		
	0.33	22.98 ± 3.55		
Tryptophan	16.67	37.51 ± 2.93	-	-
Orientin	8.33	72.55 ± 4.10	5.59 ± 0.42	12.47 ± 0.95
	3.33	31.76 ± 3.19		
	1.67	17.56 ± 2.21		
Isoorientin	8.33	60.92 ± 2.81	6.53 ± 0.53	14.55 ± 1.18
	3.33	31.69 ± 4.94		
	1.67	17.87 ± 3.08		
Vitexin	16.67	46.17 ± 2.65	-	-
Isovitexin	16.67	44.40 ± 4.91	-	-
Luteolin-7-*O*-glucoside	8.33	52.11 ± 3.98	8.00 ± 0.68	17.84 ± 1.51
	3.33	22.66 ± 2.47		
	1.67	13.49 ± 1.09		
Luteolin-7-*O*–rutinoside	16.67	79.82 ± 0.67	9.38 ± 0.47	15.77 ± 0.80
	8.33	49.55 ± 3.91		
	3.33	21.44 ± 1.46		
Rosmarinic acid	8.33	60.25 ± 4.66	6.91 ± 0.61	19.18 ± 1.69
	3.33	23.61 ± 2.27		
	1.67	16.20 ± 3.85		
1-*O*-Feruloyl-d-glucoside	16.67	59.10 ± 2.55	13.31 ± 0.23	37.35 ± 0.64
	8.33	35.36 ± 1.54		
	3.33	13.51 ± 1.62		
1-*O*-Caffeoyl-d-glucoside	8.33	50.87 ± 1.30	8.04 ± 0.49	23.47 ± 1.43
	3.33	29.84 ± 1.19		
	1.67	14.07 ± 0.39		

Inhibition rate was calculated as percentage with respect to the control value. The IC_50_ values of each sample were estimated from the least-squares regression line of the logarithmic concentration plotted against inhibitory activity. ^a^ Trolox and ^b^ quercetin were used as positive controls. Inhibitory effect was expressed as mean ± SD of triplicate experiments.

## 3. Experimental

### 3.1. Chemicals and Reagents

dl-Glyceraldehyde, the reduced form of nicotinamide adenine dinucleotide phosphate (NADPH), bovine serum albumin (BSA), sodium phosphate and quercetin used in this study were purchased from Sigma (St. Louis, MO, USA). Human recombinant aldose reductase was purchased from Wako Pure Chemical Industries (Osaka, Japan). All other chemicals and reagents used were of analytical grade.

### 3.2. Plant Materials

*Colocasia esculenta* (L.) Schott was purchased from Dae Kwang Herb Medicine Co., Ltd. (Chuncheon, Korea) and the voucher specimen (No. RIC-1021) was deposited at Regional Innovation Center, Hallym University, Republic of Korea.

### 3.3. Extraction and Isolation

Fresh *Colocasia esculenta* (L.) Schott (7.3 kg) leaves were extracted with 95% ethanol (60 L × 2 times) for 2 h at 100 °C. The combined filtrates were concentrated to dryness *in* *vacuo* at 40 °C. The extract was suspended in distilled water and partitioned sequentially with *n*-hexane, methylene chloride (CH_2_Cl_2_), ethyl acetate (EtOAc) and *n*-butanol (BuOH), respectively. The EtOAc fraction showed strong inhibitory activity on AR, so this fraction (7 g) was chromatographed over a Sephadex LH-20 column using MeOH–H_2_O (1:1, v/v) as eluent to afford eight pooled fractions (CLEF 1-8). Fractions CLEF 1 and 2 were further fractionated using Sephadex LH-20 column chromatography and MeOH–H_2_O (4:1, v/v) as eluent to give compounds **5** (26.1 mg), **3** (6.8 mg) and **8** (10.9 mg). Fractions CLEF 4 and 5 were subjected to Sephadex LH-20 column chromatography with MeOH–H_2_O (1:1, v/v) as the eluent to yield eight subfractions (CLEF 4-5:Fr. 1 to 8). Fraction CLEF 4:Fr. 1 was chromatographed on a Sephadex LH-20 column with MeOH–H_2_O (1:2, v/v) to give compound **6** (13.6 mg) and fraction CLEF 5:Fr. 6 was further fractionated using Sephadex column chromatography and MeOH–H_2_O (4:1, v/v) to give compound **9** (10.0 mg). The BuOH fraction also showed AR inhibitory activity, so this fraction (73 g) was further purified by using a Diaion HP-20 column and a Sephadex LH-20 column; elution of the Sephadex LH-20 column with MeOH–H_2_O mobile phases (1:1, 2:3, 3:7); resulted in the isolation of compounds **2** (28.6 mg), **4** (53.2 mg) and **1** (61.9 mg), **10** (28.4 mg) and **7** (52.9 mg).

### 3.4. Preparation of Aldose Reductase

Crude rat lenses aldose reductase (rAR) was prepared as follows: lenses were removed from Sprague-Dawley rats weighing 250–280 g and frozen at −70 °C until use. The rat lens homogenate was prepared according to the method of Hayman and Kinoshita with some modifications [33,34,35]. Non-cataractous transparent lenses were pooled and homogenate was prepared in 0.1 M phosphate buffer saline (pH 6.2). After centrifugation at 10,000 rpm for 20 min in a refrigerated centrifuge, the supernatant, which was then collected and as the rAR, all procedures were carried out at 4 °C.

### 3.5. Determination of Aldose Reductase Inhibition in Vitro

AR activity was assayed spectrophotometrically by measuring the decrease in the absorption of NADPH at 340 nm over a 4-min period according to the method of Hayman and Konoshita with some modifications, using dl-glyceraldehyde as the substrate. Each 1.0 mL cuvette contained equal units of the enzyme, 0.10 M sodium phosphate buffer (pH 6.2), 0.3 mM NADPH, with or without 10 mM of the substrate and an inhibitor [10,36]. The concentration of inhibitors giving 50% inhibition of enzyme activity (IC_50_) calculated from the least-squares regression line of the logarithmic concentrations plotted against the residual activity.

### 3.6. Kinetics of Recombinant Human Aldose Reductase

Reaction mixtures consisted of 0.1 M potassium phosphate, 0.16 mM NADPH, 2 mM of recombinant human aldose reductase (rhAR) with varied concentrations of substrate dl-glyceraldehyde and AR inhibitor in a total volume of 200 µL. Concentrations were ranged from 0.1 to 1 mM for dl-glyceraldehyde, from 0.1 to 1 mM for active compound. Recombinant human aldose reductase activity was assayed spectrophotometrically by measuring the decrease in absorption of NADPH at 340 nm after substrate addition using a Bio Tek Power Wave XS spectrophotometer (Bio Tek Instruments, Winooski, VT, USA) [34]. 

### 3.7. Lens Culture and Intracellular Galactitol Measurement

Lenses isolated from 10-week-old male rats were cultured for 6 days in TC-199 medium that contained 15% fetal bovine serum 100 units/mL penicillin and 0.1 mg/mL streptomycin under sterile conditions in an atmosphere of 5% CO_2_ and 95% air at 37 °C. Samples were dissolved in dimethyl sulfoxide. The lenses were divided into five groups and cultured in medium containing 5 mM glucose, 30 mM galactose and rosmarinic acid or caffeic acid ethylene ester. Each lens was placed in well containing 1.0 mL of medium. Galactitol was determined by HPLC after its derivatization by reaction with benzoic acid to a fluorescent compound [37].

### 3.8. Blood Culture and Intracellular Galactitol Measurement

Blood sample was collected in heparin containing polypropylene tube from 10-week-old male rats. For sugar and sugar alcohol analysis, erythrocytes from heparinized blood were separated from the plasma and buffy coat by centrifuging at 2000× *g* for 10 min. The cells were washed thrice with normal saline (0.9% NaCl) at 4 °C. In the final washing, the cells were centrifuged at 2000× *g* for 10 min to obtain a consistently packed cell preparation. The packed cells (1 mL) were then incubated in a Krebs-Ringer bicarbonate buffer (pH 7.4) containing 30 mM galactose in the presence or absence of samples at 37 °C in 5% CO2 for 3 h. The erythrocytes were washed with cold saline by centrifuging at 2000× *g* for 10 min, precipitated by adding 6% of cold perchloric acid (3 mL), and centrifuged again at 2000× *g* for 10 min. The supernatant was neutralized with 2.5 M K_2_CO_3_ at 4 °C and used for galactitol determination [38]. HPLC analysis for sugar and sugar alcohol in blood was performed with this supernatant of red blood cell homogenate after being benzoylated.

### 3.9. ABTS^+^ Assay

The method of Re *et al.*, [39] was used with slight modifications. ABTS diammonium salt (2 mM) and potassium persulfate (3.5 mM) were mixed, diluted in distilled water and kept in the dark at room temperature for 24 h before use. After addition of ABTS^+^ solution to 10 µL of antioxidant compounds were recorded at after 10 min reaction. The percentage inhibition of absorbance at 750 nm is calculated and potted as a function of concentration of antioxidants. Trolox was used as positive control.

## 4. Conclusions

The present study isolated ten compounds from the leaves of *C. esculenta*. Among the isolated compounds orientin (**2**) and isoorientin (**3**) significantly inhibited rat lens aldose reductase with IC_50_ values of 1.65 and 1.92 μM, respectively. Specifically, the inhibitory activity of compound **2** was 3.9 times greater than that of the positive control (quercetin = 4.12 μM). Kinetic analysis using Lineweaver-Burk plots of 1/velocity and 1/concentration indicate that compounds **2** and **3** are noncompetitive inhibitors. Our results indicate that flavonoids **2** and **3** isolated from *C. esculenta* leaves prevented the accumulation of sorbitol in rat lenses. On the basis of AR inhibition, we conclude that compounds **2** and **3** have therapeutic potential for preventing and treating diabetic complications, although further clinical research is needed. 
